# The Dark Triad, Moral Disengagement, and Social Entrepreneurial Intention: Moderating Roles of Empathic Concern and Perspective Taking

**DOI:** 10.3389/fpsyg.2020.01520

**Published:** 2020-07-28

**Authors:** Wenqing Wu, Yuzheng Su, Xuan Huang, Wenyi Liu, Xin Jiang

**Affiliations:** ^1^College of Management and Economics, Tianjin University, Tianjin, China; ^2^Business School, University of Leeds, Leeds, United Kingdom; ^3^Renmin Business School, Renmin University of China, Beijing, China; ^4^School of International Education, Tianjin University, Tianjin, China

**Keywords:** social entrepreneurial intention, dark triad, moral disengagement, empathic concern, perspective taking

## Abstract

Past research about social entrepreneurial intention has centered on the impact of bright personalities; however, dark personalities such as the dark triad are also considered to have advantages. This study explored the relationship between the dark triad and social entrepreneurial intention by focusing on the mediating role of moral disengagement and the moderating role of empathic concern and perspective taking. Based on a sample of 491 undergraduates and 412 students in a master in business administration program in China, the dark triad was found to be negatively related to social entrepreneurial intention through moral disengagement. Moreover, high levels of empathic concern and perspective taking weakened the direct effect of the dark triad on moral disengagement, as well as the indirect effect of the dark triad on social entrepreneurial intention. Our study extends the research in the field of personality and entrepreneurship. Given the findings on the role of moral disengagement, empathic concern, and perspective taking, education efforts may assist in decreasing the negative effects of the dark triad on social entrepreneurial intention.

## Introduction

Social entrepreneurial intention (SEI) is gaining momentum in entrepreneurial research (Bandura et al., 1996; Liñán and Fayolle, 2015). Unlike business entrepreneurial intention (EI), which means to create a business for the purpose of profitability, SEI refers to the willingness of pursuing social missions by establishing a social venture (Bacq and Alt, 2018). Social entrepreneurs view social issues from a business perspective and use commercial planning to solve social problems, such as poverty, disease, and so on. And practice has proved that social enterprises have played an important role in helping solve social problems (Wu et al., 2020). According to Brockhaus, who applied the theory of personality in the field of entrepreneurship, identifying that personality traits of entrepreneurs should be the key topic of entrepreneurship studies (Brockhaus Sr, 1980). Recent studies have confirmed that generally positively perceived personality such as self-efficacy (Hockerts, 2017; Fuller et al., 2018) and compassion (Miller et al., 2012) has significant impacts on SEI. In fact, all personality traits, whether bright or dark, may provide advantages and disadvantages for entrepreneurs (Smith et al., 2018). Even dark personality traits such as narcissism and aggressiveness are needed in certain situations to help overcome difficulties such as raising funds during start-up period (Miller, 2015). Therefore, the role of the dark triad (DT) – which is the combination of the personality traits of Machiavellianism, psychopathy, and narcissism (Paulhus and Williams, 2002)—is valuable in the field of social entrepreneurship research (Do and Dadvari, 2017).

According to the theory of planned behavior, intentions can effectively and reliably predict individual behavior (Ajzen, 1991; Urban and Chantson, 2019). Individuals with SEI, to a large extent, represent that they seek to implement social entrepreneurial activities to help address society’s unmet needs. Actually, DT has been identified to have positive social outcomes such as acquiring to important resources and benefiting the organization (Do and Dadvari, 2017; Smith et al., 2018). However, SEI combines not only commercial purpose but also social purpose, and the impact of DT on SEI is greatly ignored. Therefore, the specific question we first seek to explore in this research is whether the impact of DT on SEI is positive and how DT affects SEI.

Moreover, social cognitive theory has identified a promising mediator, moral disengagement (MD), which explains how people avoid feeling guilty after engaging in harmful behavior that does not conform to their moral code (Bandura et al., 1996; Bandura, 1999). In prior researches, DT was confirmed to correlate with low scores of conscientiousness (Paulhus and Williams, 2002; Jones et al., 2017), which may easily lead to MD. In addition, MD is also regarded as a predictor of antisocial behavior (Bandura et al., 2001), and thus individuals with MD may show lower levels of intention in the face of social entrepreneurship.

**FIGURE 1:**
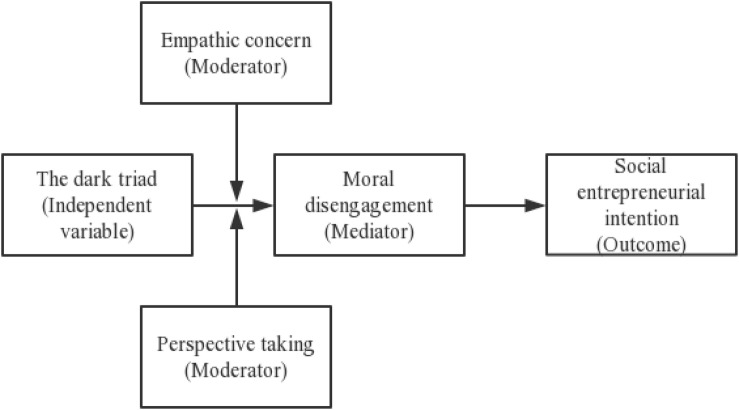
The heuristic relationship of the study.

In addition, MD does not exist as a fixed characteristic, but rather influenced by both social features, as well mental activities (Bandura, 2002b). Detert et al. (2008) found that people who were more empathic were more likely to refrain from MD. Thus, empathy is a promising factor that may inhibit MD. However, empathy is a complex phenomenon, involving at least two fundamental components: empathic concern (EC) and perspective taking (PT) (Wakabayashi et al., 2006; Greenberg et al., 2015); it has been recommended that researchers consider different aspects of empathy to obtain more accurate inferences (Schalkwijk et al., 2016). Hence, the third step of this research is to separately check the moderating role of the two aspects of empathy, EC and PT. The heuristic relationship of our study is shown in Figure 1.

## Theory and Hypotheses

### The Dark Triad and Social Entrepreneurial Intention

Personality plays an important role in situations that are complex and full of uncertainty, such as social entrepreneurial activities (Frank et al., 2007). Indeed, scholars have recently become more interested in the role of personality in the study of social entrepreneurship. In particular, DT was found to affect all domains of human behaviors (Lee et al., 2013) and even produce positive results (Hogan and Hogan, 2001), which bring new ideas to the study of SEI.

The three overlapping dimensions of DT are Machiavellianism, psychopathy, and narcissism (Paulhus and Williams, 2002). Specifically, Machiavellianism means emotional indifference toward others and the absence of traditional morality (Christie and Geis, 1970); psychopathy is represented by impulsiveness and emotional indifference (Lilienfeld and Andrews, 1996), and narcissism is defined by self-centeredness, admiration of vanity, and the enjoyment of superiority (Raskin and Hall, 1979). All three dimensions are considered socially aversive personality traits (Paulhus and Williams, 2002). Wu et al. (2019a, b) believed that individuals with DT prefer to gain higher returns in a short time, so as to participate in behaviors that neglect social responsibility such as destroying the environment, which violated the mission of social entrepreneurship.

It is worth noting that people are more likely to generate in entrepreneurial activities if they scored high in DT (Do and Dadvari, 2017). However, Kramer et al. (2011) explain that their passion for entrepreneurship may be based on selfish competitive strategies instead of true desire. SEI arises from a high degree of concern toward social issues through an intention to help others, which largely circumvents self-serving strategies. Therefore, unlike the positive relationship between DT and EI (Do and Dadvari, 2017; Wu et al., 2019b), the influence of DT on SEI may not be positive. Based on the above, we propose Hypothesis 1:

**Hypothesis 1:** DT is negatively related to SEI.

### Moral Disengagement as a Mediator

According to Garriga and Melé (2004) and Ven et al. (2010), entrepreneurs increasingly deem bearing social responsibilities to be an ethical duty. As a “subspecies” of the entrepreneur group (Dees, 1998), social entrepreneurs are more likely to view social responsibilities as their moral obligation. As underlined by the concept of MD, most people will behave morally when moral self-regulation is activated, but moral standards are not a stable constraint but rather can be selectively deactivated, which is known as MD. Moral disengagement is triggered by eight correlative cognitive mechanisms, including euphemistic labeling, dehumanization, displacement of responsibility, and so on. Studies have shown that MD can affect externalization attitudes of individuals (Muratori et al., 2017), and it has been found that MD can mediate the relationship between DT and immoral consumer attitudes (Egan et al., 2015). Considering that the mechanism of MD can serve as a means to avoid social responsibility, we expect that DT may affect individual attitude toward social entrepreneurship through MD.

Specifically, MD often leads to negative behaviors such as general unethical behavior and a series of negative psychological states, including increasing the dehumanization of others (Moore, 2015). Moreover, individuals with MD usually make self-serving decisions (Bandura et al., 2001; Moore et al., 2012) and engage in less prosocial behavior (Bandura et al., 2001), such as responding to fewer social concerns. Indeed, studies have shown that high levels of MD are related to serious antisocial behavior in young people (Bandura et al., 2001; Gini, 2006; Shulman et al., 2011). Therefore, it appears that individuals with MD may have an extremely indifferent attitude toward social issues, which result in deceased SEI.

Further, Paulhus and Williams (2002), Li-Ping Tang et al. (2008), Kish-Gephart et al. (2010) highlighted that MD is based on two components of DT: Machiavellianism and psychopathy. First, Machiavellian individuals show more ruthlessness and selflessness in their daily work than psychopathic and narcissistic individuals. The calculative aspect makes them ignore moral norms in the face of moral dilemmas, which may easily lead to MD. Second, individuals with Machiavellian and psychopathic traits have lower scores of conscientiousness (Paulhus and Williams, 2002), which makes them easily abandon self-discipline. Regarding narcissism, Hoffman et al. (2011) opined that narcissistic leaders tend to displace responsibility: that is, attributing success and development of the group to themselves while attributing bad outcomes to the group. In addition, callousness is a conjoint core among the components of DT (Jones and Paulhus, 2011) and also a component of MD (Hyde et al., 2010; Muratori et al., 2017). Previous research has confirmed that young people with higher callousness may be more likely to implement MD (Shulman et al., 2011).

Thus, it appears that DT is closely related to MD, which in turn leads to decreased SEI. Therefore, we propose the following as Hypothesis 2:

**Hypothesis 2:** Moral disengagement mediates the relationship between DT and SEI.

### Empathic Concern and Perspective Taking as Moderators

Mair and Noboa (2006) identified several unique aspects of the traditional measures used in the theory of planned behavior and EI model, including not only moral obligation as representative of social norms but also empathy as representative of the attitude toward behavior. This idea has been applied to the international context by scholars (Tukamushaba et al., 2011), while keeping all variables and assumptions from Mair and Noboa’s model.

First, as the affective component of empathy, EC means feeling compassion and care for less fortunate people based on their perspective (Davis, 1983), and EC is considered to contribute to moral judgments (Decety et al., 2011). Having concern for the suffering of others makes individuals more likely to behave consistent with their moral standards, and they will suffer from guilt and self-condemnation when they betray those standards (Bussey et al., 2015), thus inhibiting MD. Additionally, high levels of EC have been found to be related to high moral cognition and moral cognation processes (Pohling et al., 2016). In short, highly empathic people are able to put themselves in the shoes of others easier and therefore avoid experiencing MD (Greenberg et al., 2015).

Second, as the cognitive component of empathy, PT refers to the tendency to understand others’ psychological views (Davis, 1983), and PT is also considered to contribute to moral judgments (Decety et al., 2011). Bergman argued that thinking from the viewpoints of others is beneficial for understanding the impact of behavior on others, thus enhancing ethical perception and consciousness (Bergman, 2002). Moreover, managers were found to possess a higher sense of responsibility toward others, as well as a high degree of moral awareness, and when they adopt others’ viewpoints at work, it likely provides benefits to the whole organization (Mencl and May, 2009). Thus, it is speculated that DT individuals with high PT will engage in less MD. On the basis of the above, we propose that:

**Hypothesis 3a:** Empathic concern moderates the relationship between DT and MD, such that the relationship is weaker when EC is higher.
**Hypothesis 3b:** Perspective taking moderates the relationship between DT and MD, such that the relationship is weaker when PT is higher.

The above hypotheses represent an integrated framework in which MD mediates the negative relationship between DT and SEI, and EC and PT moderate the positive relationship between DT and MD. Further, a previous study has found that EC and PT can arouse prosocial intentions (Batson, 2014), and indeed EC and PT are believed to facilitate prosocial behavior (Habashi et al., 2016; Guo et al., 2019), which is considered a driving factor in individuals contemplating engagement in social entrepreneurship (Bacq and Alt, 2018). Hence, it is logical to suggest that EC and PT also moderate the strength of the mediating mechanism for MD in the relationship between DT and SEI. That is, the indirect effect of DT on SEI may be weaker for individuals high in EC or PT. However, when individuals’ EC or PT levels are low, DT may be more influential on SEI. Taken together, we propose that:

**Hypothesis 4a:** Empathic concern moderates the indirect effect of DT on SEI via MD so that the indirect effect is weaker when EC is higher.
**Hypothesis 4b:** Perspective taking moderates the indirect effect of DT on SEI via MD so that the indirect effect is weaker when PT is higher.

## Materials and Methods

### Participants and Procedures

It has been suggested that to measure SEI more accurately, people facing professional decision-making are suitable research samples (Hockerts, 2017). Thus, to survey our proposed hypotheses, we used an undergraduate student sample for Study 1. In addition, considering that students with SEI are not limited to business majors (Krueger and Carsrud, 1993; Hmieleski and Lerner, 2016), particularly in China where entrepreneurship is strongly encouraged nowadays, we also included students from other majors such as the arts, sciences, and engineering in our sample. Besides, to check the robustness of the results, we conducted Study 2 using a student sample of graduate students enrolled in a master in business administration (MBA) program. If the results of Study 2 are consistent with Study 1, then the robustness of our research is satisfied. In addition, we have to declare that we have obtained the informed consent of the participants before the investigation.

For Study 1, which was conducted in December 2018, students from 13 universities in Tianjin, China, participated in the survey. We distributed 800 questionnaires through the Internet and offline channels, and 615 undergraduates returned the survey, indicating a response rate of 77%. However, some surveys were excluded because they were not completely answered; we finally obtained 491 respondents, with an effective response rate of 80%. As detailed in Table 1, 48% of our sample was male, and 52% was female, and the average age was 22 years. Forty-two percent of participants reported that they had entrepreneurial practice experience, and 47% of participants reported that their family members had formed sole proprietorships or partnerships.

**TABLE 1:** Mean, standard deviation, Pearson zero-order correlation, and the discriminant validity test for Study 1.

Variable	Mean	SD	1	2	3	4	5	6	7	8	9	10	11
Gender	1.52	0.50											
Age	22.00	2.54	–0.06										
Family business background	1.58	0.50	0.04	–0.09									
Past entrepreneurial practice experience	1.53	0.50	0.02	−0.15**	0.33**								
Machiavellianism	3.74	1.11	−0.14**	0.03	−0.30**	−0.36**	**0.83**						
Psychopathy	3.72	1.07	–0.06	0.09	−0.37**	−0.41**	0.77**	**0.80**					
Narcissism	4.38	0.79	–0.01	−0.12*	–0.01	0.01	0.36**	0.22**	**0.71**				
MD	3.53	1.16	−0.11*	0.19**	−0.37**	−0.48**	0.73**	0.74**	0.10*	**0.81**			
EC	4.75	0.81	0.06	–0.04	0.26**	0.32**	−0.46**	−0.53**	0.01	−0.52**	**0.75**		
PT	4.75	0.81	0.04	−0.11*	0.28**	0.32**	−0.47**	−0.50**	0.04	−0.58**	0.66**	**0.76**	
SEI	4.51	0.79	–0.06	–0.01	0.13**	0.13**	−0.27**	−0.31**	0.07	−0.31**	0.44**	0.49**	**0.77**

*N* = 491; *p < 0.05 and **p < 0.01 (two-tailed). Numbers in bold represent AVE.

### Measures

Questionnaire for Study 1 contained a total of 32 items, measured by a seven-point Likert scale ranging from 1 (strongly disagree) to 7 (strongly agree), with 4 being a neutral response. The original scales were in English, and thus we first translated them into Chinese and back-translated into English to ensure accuracy. Furthermore, the average score for all items related to each variable was used, with higher scores reflecting higher levels of the variable measured.

To evaluate the internal reliability, convergent validity, and discriminant validity of our scales, first, in line with (Flatten et al., 2015; Bacq and Alt, 2018), the Cronbach α and composite reliability of each scale were applied to test the internal reliability, using 0.6 as the recommended cutoff. Next, to test the convergent validity of our scales, the average variance extracted (AVE) was applied, using the acceptance criterion of 0.5, which means that the variable explains at least half of the variance of its indicators. Finally, to ensure discriminant validity, the square root of the AVE of each variable was calculated according to Claes and Larcker (1981), following the guideline that it should be greater than the Pearson zero-order correlation between the corresponding constructs.

#### The Dark Triad

The 12-item Dirty Dozen scale was applied to measure DT (Jonason and Webster, 2010), with each of the three dimensions consisting of four items. An example item of Machiavellianism is “I have used deceit or lied to get my way”; the Cronbach α was 0.85, and the AVE was 0.69. An example item of psychopathy is “I tend to lack remorse”; the Cronbach α was 0.81, and the AVE was 0.64. An example item of narcissism is “I tend to want others to admire me”; the Cronbach α was 0.66, and the AVE was 0.50. In addition, the composite reliabilities of the three dimensions exceeded 0.80. Thus, the internal reliability, convergent validity, and discriminant validity of all dimensions of this scale were confirmed.

#### Moral Disengagement

Moral disengagement was measured with an eight-item scale constructed by Moore et al. (2012), which is particularly adapted for a wide range of adult participants, and the eight-item scale was confirmed to have no differences from those complex scales both on statistical and practical grounds (Moore et al., 2012). Example items of the scale are euphemistic labeling: “Taking something without the owner’s permission is okay as long as you’re just borrowing it”; and dehumanization: “Some people have to be treated roughly because they lack feelings that can be hurt.” The Cronbach α of the scale in our study was 0.92, the composite reliability was 0.94, and the AVE was 0.65, confirming reliability and validity.

#### Empathic Concern and Perspective Taking

We assessed EC and PT using two scales recommended by Davis (1980), consisting of four and five items, respectively. “I often have tender, concerned feelings for people less fortunate than me” is an example of an item measuring EC. The EC items obtained a Cronbach α of 0.75 in our study, a composite reliability of 0.84, and an AVE of 0.57. “I sometimes try to understand my friends better by imagining how things look from their perspective” is an example of an item measuring PT. The PT items obtained a Cronbach α of 0.82 in our study, a composite reliability of 0.87, and an AVE of 0.58. Thus, these scales were reliable and valid.

#### Social Entrepreneurial Intention

SEI was assessed using a three-item scale, which was a combination of the adjusted version of previous EI scales (Douglas and Shepherd, 2002; Thompson, 2009; Hockerts, 2017) and an item adapted from the EI scale used by Liñán and Chen (2009). For example, “I expect that at some point in the future I will be involved in launching an organization that aims to solve social problems.” In this study, the Cronbach α of the scale was 0.67, the composite reliability was 0.82, and the AVE was 0.60, indicating reliability and validity.

#### Control Variables

Previous research has highlighted that gender differences have a significant impact toward entrepreneurship activities (Laudano et al., 2019). In addition, recognizing that students’ intention toward social entrepreneurship may change with age (Hatak et al., 2015), we added gender (0 = male, 1 = female) and age as control variables. Further, family business background (0 = yes, 1 = no) and past entrepreneurial practice experience (0 = yes; 1 = no) were also controlled because students with family or personal entrepreneurial experience may better understand the entrepreneurial process, which may influence SEI (Chlosta et al., 2012; Vaillant and Lafuente, 2019).

### Data Analysis

To test our hypotheses about the mechanism of how DT influences SEI, SPSS, Bizinsight (Beijing) Information Technology Co., Ltd., Beijing, China was used. First, as explained above, we confirmed the quality of our scales including their internal consistency reliability and convergent and discriminant validity. Second, we tested the main effect by multiple regression analysis. Third, MacKinnon’s four-step method (MacKinnon, 2012), a widely used method of mediation test, was applied to test the mediation effect in our research. Fourth, multiple regression analysis was applied again to test the moderation effect. Furthermore, we evaluated whether the mediation effect was moderated by EC and PT using path analysis with PROCESS macro as suggested by Hayes (2017). After completing these procedures, we repeated the same analyses using our sample of MBA students (Study 2) to ensure the robustness of our results.

## Results

### Test of Hypotheses

As detailed previously, our scales showed a satisfactory level of internal reliability and met the standards in both convergent and discriminant validity. The descriptive statistics of our sample are shown in Table 1.

To prove Hypothesis 1 – that DT was negatively related to SEI – we constructed Models 1 and 2 (Table 2) using SEI as the dependent variable. Initially, we only input control variables into Model 1. Next, the independent variable was added to Model 2, which revealed a significant negative relationship between DT and SEI, *b* = −0.21, *p* < 0.001. Thus, Hypothesis 1 was supported.

**TABLE 2:** Results of multiple regression analysis in Study 1.

Variables	Social entrepreneurial intention				Moral disengagement			
		
	Model 1	Model 2	Model 3	Model 4	Model 5	Model 6	Model 7	Model 8
Gender	–0.10	–0.13	−0.15*	−0.15*	−0.20*	–0.08	–0.08	–0.10
Age	–0.00	–0.00	0.01	0.01	0.05**	0.06***	0.05***	0.05***
Family business background	0.16*	0.08	0.04	0.03	−0.54***	−0.25**	0.20**	−0.16*
Past entrepreneurial practice experience	0.16*	0.07	–0.04	–0.04	−0.89***	−0.52***	−0.42***	−0.40***
DT		−0.21***		–0.04		0.84***	1.23***	1.45***
MD			−0.23***	−0.21***				
EC							0.04	
PT								0.11
DT * EC							−0.10*	
DT * PT								−0.15***
R^2^	0.03	0.07	0.11	0.11	0.30	0.58	0.62	0.65
Adjusted *R*^2^	0.02	0.06	0.10	0.10	0.29	0.58	0.62	0.65
*F*	3.74**	7.04***	11.38***	9.53***	51.7***	135.44***	112.60***	128.19***

*N* = 491; *p < 0.05, **p < 0.01, ***p < 0.001 (two-tailed).

In Hypothesis 2, we anticipated that MD plays a mediating role in the relationship between DT and SEI. To test this hypothesis, the four-step method of MacKinnon (2012) was applied, which requires (a) a significant relationship between DT and SEI, (b) a significant relationship between DT and MD, (c) a significant relationship between MD and SEI when DT is controlled, and (d) a significant coefficient of the indirect path between DT and SEI via MD. Whether the last requirement is satisfied depends on the result of the bias-corrected percentile bootstrap.

As Table 2 shows, (a) DT was negatively related to SEI, *b* = −0.21, *p* < 0.001 (Model 2); (b) DT had a significantly positive association with MD, *b* = 0.84, *p* < 0.001 (Model 6); (c) MD had a significantly negative association with SEI after DT was controlled, *b* = −0.21, *p* < 0.001 (Model 4); and (d) the indirect relationship between DT and SEI via MD was significant (*b* = −0.17, SE = 0.05, 95% confidence interval [CI] = [−0.28, −0.08]). Overall, the four standards for establishing a mediation effect were fully satisfied, which revealed that MD mediated the relationship between DT and SEI. Thus, Hypothesis 2 was supported. More importantly, in Model 4, we can see that the direct impact of DT on SEI became non-significant compared with Model 2, indicating that MD completely mediated the relationship between DT and SEI.

In Hypotheses 3a and 3b, we expected that both EC and PT moderate the relationship between DT and MD. As Table 2 illustrates, in Model 7, DT ^∗^ EC was significantly related to MD (*b* = −0.10, *p* < 0.05). In Model 8, DT ^∗^ PT was also significantly related to MD (*b* = −0.15 *p* < 0.001). Thus, the relationship between DT and MD was moderated by EC and PT, supporting Hypotheses 3a and 3b.

**FIGURE 2:**
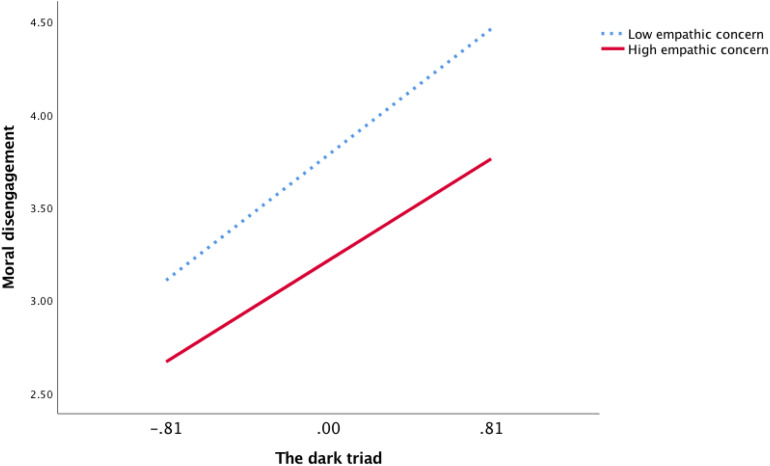
The moderation effect of empathic concern in Study 1.

**FIGURE 3:**
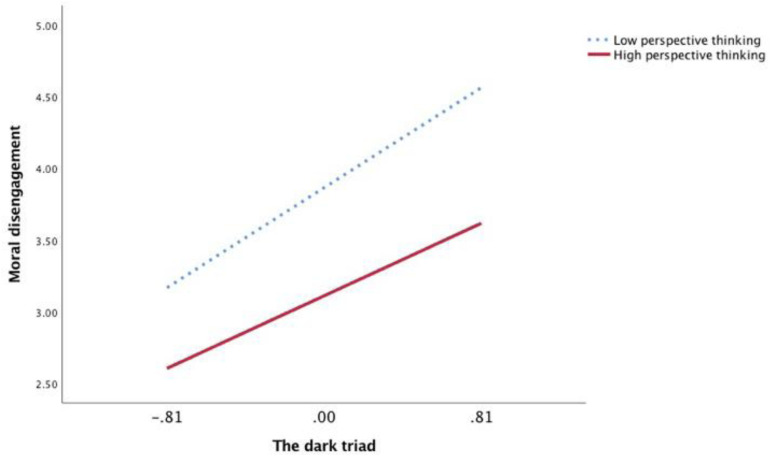
The moderation effect of perspective taking in Study 1.

Furthermore, to show the moderating role of EC and PT vividly, we plotted the regression of DT on MD for high (1 standard deviation above the mean) and low (1 standard deviation below the mean) levels of EC and PT using simple slope tests (Figures 2, 3). As shown in Figure 2, there was a significantly positive relationship between DT and MD at low levels of EC (*b* = 0.84, *p* < 0.001). However, the relationship between DT and MD was weaker at high levels of EC (*b* = 0.68, *p* < 0.001). Similarly, as shown in Figure 3, the relationship between DT and MD was also weaker at high levels of PT (*b* = 0.63, *p* < 0.001) than at low levels of PT (*b* = 0.87, *p* < 0.001). Thus, Hypotheses 3a and 3b were further supported.

In Hypotheses 4a and 4b, we expected that EC and PT would moderate the indirect path of DT on SEI via MD. To test the moderated mediation effect, we performed path analysis with Hayes’ PROCESS, bootstrapping 5,000 samples to compute bias-corrected CIs. The results demonstrated that the indirect effect of DT on SEI via MD was moderated by both EC and PT. First, for low EC individuals, a significant negative indirect relationship between DT and SEI was obtained, *b* = −0.17, SE = 0.05, 95% CI = [−0.28, −0.08]. This relationship was weaker for high EC individuals, *b* = −0.14, SE = 0.04, 95% CI = [−0.23, −0.06]. Also, the indirect relationship between DT and SEI was also weaker for high PT individuals (*b* = −0.13, SE = 0.04, 95% CI = [−0.21, −0.06]) than for low PT individuals (*b* = −0.18, SE = 0.05, 95% CI = [−0.28, −0.08]). Thus, Hypotheses 4a and 4b were supported in that DT interacts with EC and PT to influence MD, which in turn influences SEI. Given that EC and PT, respectively, moderated the first half of the mediation procedure, we refer to it as a “first stage moderation model” (Edwards and Lambert, 2007).

### Robustness Test

To ensure the robustness of our results, we conducted Study 2. First, we replaced our sample with MBA students from Tianjin, China, because MBA students with excellent sustainability concepts have been increasingly favored by employers recently (Awaysheh and Bonfiglio, 2017). We distributed 500 questionnaires in the classroom, and all of them were returned. However, 88 questionnaires were excluded because of incomplete information, providing an effective response rate of 82%. The average age of respondents in this study was 31. Next, the measurement of SEI was replaced by another version created by Bacq and Alt (2018). Lastly, the same standards of internal reliability and convergent and discriminant validity used in Study 1 applied in Study 2. If the results of Study 2 are consistent with Study 1, then the robustness of our research is satisfied.

The means, standard deviations, and correlations of Study 2 are shown in Table 3. As shown in Table 4, Study 2 also met the standards of internal reliability, convergent validity, and discriminant validity. We followed the same procedure as Study 1 to test our hypotheses.

**TABLE 3:** Mean, standard deviation, Pearson zero-order correlation, and the discriminant validity test of Study 2.

Variable	Mean	SD	1	2	3	4	5	6	7	8	9	10	11
Gender	0.52	0.50											
Age	30.66	3.99	−0.16**										
Family business background	0.27	0.45	–0.04	–0.07									
Past entrepreneurial practice experience	0.19	0.39	0.15**	−0.15**	0.40**								
Machiavellianism	2.82	1.32	−0.18**	0.10*	–0.00	–0.00	**0.80**						
Psychopathy	2.72	1.16	−0.22**	0.02	–0.01	–0.03	0.68**	**0.73**					
Narcissism	4.40	1.20	−0.14**	–0.06	–0.03	–0.02	0.37**	0.19**	**0.74**				
MD	2.37	1.20	−0.20**	0.05	–0.05	0.00	0.62**	0.63**	0.13**	**0.75**			
EC	5.49	1.00	0.02	–0.07	0.02	–0.01	−0.34**	−0.39**	0.01	−0.32**	**0.78**		
PT	5.43	1.05	–0.04	–0.05	0.28**	0.04	−0.32**	−0.39**	0.01	−0.32**	0.68**	**0.80**	
SEI	4.80	1.09	–0.12	–0.07	0.13**	0.17**	−0.19**	−0.23**	0.11*	−0.21**	0.40**	0.35**	**0.75**

*N* = 412; *p < 0.05 and **p < 0.01 (two-tailed). Numbers in bold represent AVE.

**TABLE 4:** Internal reliability and convergent validity of Study 2.

Variables	Cronbach’s α	CR	AVE
DT			
Narcissism	0.72	0.83	0.54
Psychopathy	0.69	0.81	0.53
Machiavellianism	0.81	0.88	0.64
MD	0.89	0.91	0.57
EC	0.78	0.86	0.61
PT	0.85	0.90	0.63
SEI	0.83	0.88	0.56

First, as Table 5 shows, (a) DT was negatively related to SEI, *b* = −0.19, *p* < 0.01 (Model 2); (b) DT had a significantly positive association with MD, *b* = 0.73, *p* < 0.001 (Model 6); (c) MD had a significantly negative association with SEI after DT was controlled, *b* = −0.18, *p* < 0.01 (Model 4); and (d) the indirect relationship between DT and SEI through MD was significant (*b* = −0.13, SE = 0.04, 95% CI = [−0.22, −0.05]). Similar to Study 1, the direct effect of DT on SEI was non-significant compared with Model 2. Overall, the full mediation effect was also established in the sample of MBA students in that MD fully mediated the relationship between DT and SEI. On the basis of the above, Hypotheses 1 and 2 were supported.

**TABLE 5:** Results of multiple regression analysis in Study 2.

Variables	Social entrepreneurial intention				Moral disengagement			
		
	Model 1	Model 2	Model 3	Model 4	Model 5	Model 6	Model 7	Model 8
Gender	−0.22*	−0.31**	−0.32**	−0.34**	−0.51***	–0.17	–0.19	−0.20*
Age	0.01	–0.01	0.00	0.00	–0.02	–0.01	–0.01	–0.01
Family business background	0.33*	0.34**	0.30**	0.31*	–0.14	–0.16	0.16	–0.14
Past entrepreneurial practice experience	0.28	0.27	0.28	0.27	–0.00	0.05	0.00	0.02
DT		−0.19**		–0.06		0.73***	1.30***	1.23***
MD			−0.21***	−0.18**				
EC							0.23	
PT								0.17
DT * EC							−0.11*	
DT * PT								−0.10*
R^2^	0.06	0.08	0.11	0.11	0.050	0.36	0.39	0.39
Adjusted *R*^2^	0.05	0.07	0.10	0.10	0.04	0.35	0.38	0.38
*F*	6.10***	7.37***	9.65***	8.19***	5.21***	45.47***	36.69***	36.97***

*N* = 412; *p < 0.05, **p < 0.01, ***p < 0.001 (two-tailed).

**FIGURE 4:**
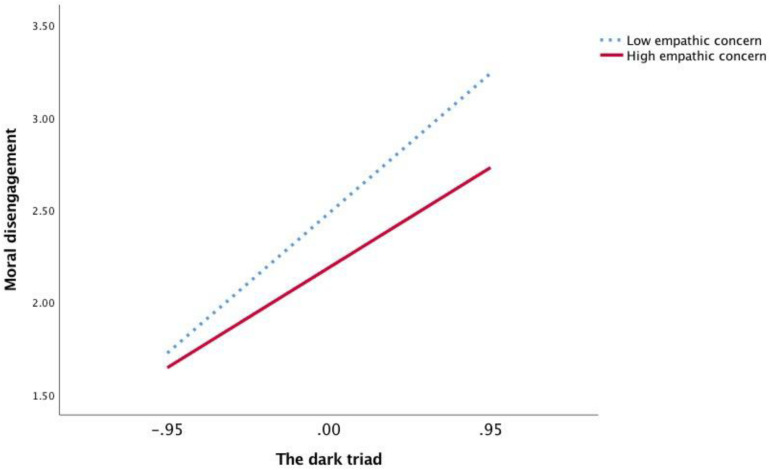
The moderation effect of empathic concern in Study 2.

**FIGURE 5:**
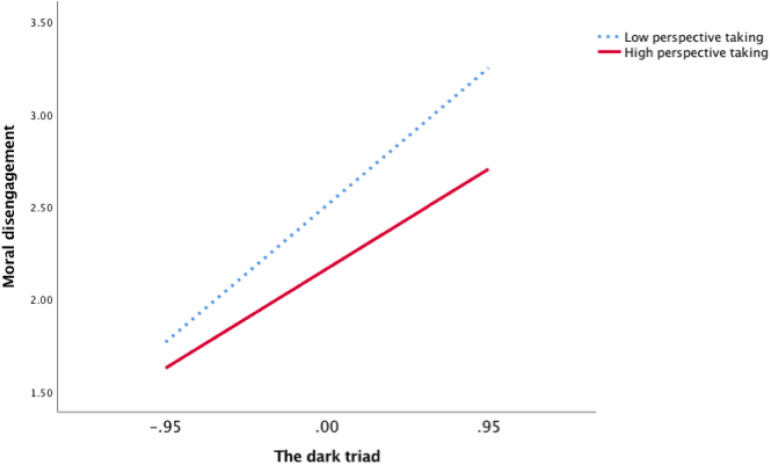
The moderation effect of perspective taking in Study 2.

Next, as Table 5 illustrates, in Model 7, DT ^∗^ EC had a significant relationship with MD (*b* = −0.11, *p* < 0.05). DT ^∗^ PT was also significantly related to MD (*b* = −0.10, *p* < 0.05). Thus, the relationship between DT and MD was moderated by EC and PT, supporting Hypotheses 3a and 3b. We also plotted the regression of DT on MD at high and low levels of EC and PT using simple slope tests, respectively (Figures 4, 5). As Figure 4 shows, a significantly positive relationship between DT and MD at low levels of EC was observed (*b* = 0.80, *p* < 0.001). However, the relationship between DT and MD was weaker at high levels of EC (*b* = 0.57, *p* < 0.001). As shown in Figure 5, the relationship between DT and MD was also weaker at high levels of PT (*b* = 0.57, *p* < 0.001) than at low levels of PT (*b* = 0.78, *p* < 0.001). Therefore, we also confirmed Hypotheses 3a and 3b.

To test the moderated mediation effect, Hayes’ PROCESS was used again. The results of the bias-corrected percentile bootstrap demonstrated that the indirect effect of DT on SEI through MD was moderated by both EC and PT, which was consistent with Study 1.

Thus, the results of Study 2 were identical to Study 1. Dark triad has a negative impact on SEI, and MD mediates the relationship. Empathic concern and PT moderate the direct relationship between DT and MD, as well as the indirect relationship between DT and SEI; that is, their direct and indirect relationship is weaker at high levels of EC or PT, and the moderation effect is even more significant compared with Study 1, indicating that the results of our research are stable and reliable.

## Discussion

Research pioneers regard personalities as the heart of entrepreneurship theory (Volkmann et al., 2012). However, with the increasing interest in social entrepreneurship, the limitations of existing studies on understanding the effects of personalities on SEI have begun to emerge. To our knowledge, no study has yet paid attention to the possible influence of DT on SEI. Therefore, in response to the call of Klotz and Neubaum (2016), we seek to construct a model to illustrate how DT affects SEI through MD, accounting for the moderating role of EC and PT. Applying a multisample research design, we obtained support for all of our hypotheses. Dark triad negatively affected SEI through MD, and the direct relation between DT and MD and the indirect relation between DT and SEI were moderated by EC and PT.

### Theoretical Contributions

First, our study extends the research in the field of personality and entrepreneurship. It was offered that successful entrepreneurship stems from personal shortcomings and disabilities (Miller, 2015), which suggests that DT may also have a positive impact on SEI. However, contrary to previous findings that show that DT is positively related to EI (Hmieleski and Lerner, 2016; Do and Dadvari, 2017), our results indicate that DT is negatively related to SEI. There are two possible reasons for this result. On the one hand, the callous nature of DT individuals may contribute to MD and a disregard of social needs. On the other hand, although individuals with DT show EI because of their competitiveness and achievement motivation, they cannot hide their lack of altruism and prosocial ideals (Hmieleski and Lerner, 2016). Indeed, their sense of achievement may be greatly discounted in the face of non–profit-oriented social entrepreneurship.

Second, we further examined the underlying mediating mechanism between DT and SEI to help explain the inner logical relationship. Beugré (2014) identified that the pursuit of social missions requires a higher level of moral engagement. Individuals acting in accordance with their moral obligations will help promote the resolution of social problems (Biljohn and Lues, 2019). However, DT showed a significantly worse moral performance (Djeriouat and Trémolière, 2014), thus leading to decreased SEI. Our finding illustrates the importance of moral cognition in cultivating SEI and are similar to those of Jones et al. (2017) and Sijtsema et al. (2019), who found that MD served as a successful mediator between DT and antisocial behavior, as well as unethical attitude in different areas. The finding also makes it possible to reduce the negative impact of DT on SEI by intervening in this path. Besides, we particularly addressed the call of Chowdhury and Fernando (2014) to further examine whether there was full or partial mediation.

Third, our results provide further support for Bandura’s (2002a) about MD. By exploring the moderating role of EC and PT, our results further indicate that the positive relationship between DT and MD is weaker under the condition of high levels of EC or PT. Further, the indirect impact of DT on SEI by way of MD is also weaker when EC or PT is high. These findings support those by Batson et al. (1997), who carried out an experiment about the effect of empathy and found that reading material full of highly empathic words is more helpful to foster volunteer intention than just reading material without such words. Additionally, these findings provide further evidence for Bandura’s theory that MD can work in some situations and not in others, making it possible to intervene in MD to improve SEI.

Moreover, our research also inspires future research toward the relationship between personality and SEI. We found that narcissism is more unique compared with Machiavellianism and psychopathy. First, as the correlation analysis shows, Machiavellianism and psychopathy have negative effects on SEI, while narcissism is slightly positively related to SEI. Although this positive correlation is not significant, it also suggests that the influence of narcissism on SEI may need further examination. Second, the three items with the lowest factor loading are all derived from items on narcissism when we performed factor analysis of DT, suggesting that narcissism may be an outlier in the three dimensions of DT. Indeed, narcissism is considered the “lightest” dimension of DT (Li-Ping Tang et al., 2008; Kish-Gephart et al., 2010; Furnham et al., 2013) and has been described as a complex personality trait with both dark and bright sides (Rogoza et al., 2018). According to Baldegger et al. (2017), narcissism can be further differentiated into narcissistic admiration and rivalry. Thus, what is the influence of the different aspects of narcissism on SEI? To obtain a more precise understanding, we suggest further study on the multiple faces of narcissism.

### Practical Contributions

In addition to the above theoretical contributions, our study has considerable practical contributions. First, because social entrepreneurs play the important role of “engines of reform” in social and economic process (Volkmann et al., 2012), this study provides a better understanding about what personal traits are of negative impact or of great importance when people plan to become social entrepreneurs and why and how the traits inhibit their thinking. Next, certain behavior is based on not only stable personality traits, but also learned reactions (Volkmann et al., 2012). Nowadays, the integration of entrepreneurship education and entrepreneurial activities has been considered a university priority (Gianiodis and Meek, 2020). Therefore, when conducting entrepreneurship education for college students, it is recommended that more courses about moral cognition and empathy be added and that well-designed training programs are implemented to foster their attention toward social needs. And teaching through social media is worth recommending, which can help students access more teaching resources about entrepreneurship (Wu and Song, 2019). Besides, a leader rotation system should be considered in campus activities to improve student’s sense of responsibility (Jones et al., 2017). Organizations and enterprises can implement policies (or enforced policies) on individuals’ responsibilities for behaviors to prevent individuals from easily breaking away from moral behavior. It is also worth thinking about conducting moral tests from time to time to remind and urge individuals to act according to moral standards. In addition, with the emphasis on people’s mental health, both colleges and enterprises can set up corresponding psychological teams to provide psychological counseling services and courses.

### Limitation and Suggestions for Future

The current study also had a number of limitations. First, although EC and PT are considered as moderators in our study, we did not include other promising moderators in the field of psychology that may strengthen or inhibit the direct and indirect relationship between DT and SEI through MD. Therefore, future research should actively explore the moderating role of psychological variables in the context of social entrepreneurship and thus provide more guidance for teachers to promote SEI. Second, our research was conducted in China, which means that the results may not be applicable in other countries. Our results should be understood in the situation of developing countries such as China, where social problems are more prominent (Hockerts, 2017). Indeed, there are considerable differences in entrepreneurial areas across countries, especially in developed countries with advanced social welfare systems (Sharma, 2015). Therefore, our findings may not translate to different social contexts, and future research about social entrepreneurship in varied cultural contexts should be pursued.

## Data Availability Statement

The raw data supporting the conclusions of this article will be made available by the authors, without undue reservation.

## Ethics Statement

Ethical review and approval was not required for the study on human participants in accordance with the local legislation and institutional requirements. The patients/participants provided their written informed consent to participate in this study. Written informed consent was obtained from the individual(s) for the publication of any potentially identifiable images or data included in this article.

## Author Contributions

All authors listed have made a substantial, direct and intellectual contribution to the work, and approved it for publication.

## Conflict of Interest

The authors declare that the research was conducted in the absence of any commercial or financial relationships that could be construed as a potential conflict of interest.
